# Movement Patterns of Juvenile Loggerhead Turtles (*Caretta caretta* L. 1758) and Green Turtles (*Chelonia mydas* L. 1758) Hatched in Captivity and Released in the Korean Waters

**DOI:** 10.3390/ani12162157

**Published:** 2022-08-22

**Authors:** Il-Hun Kim, Il-Kook Park, Dong-Jin Han, Min-Seop Kim, Daesik Park, Dae-Yeon Moon, In-Young Cho, Ji-En Im, Jaejin Park, Yong-Rock An

**Affiliations:** 1 Department of Ecology and Conservation, National Marine Biodiversity Institute of Korea, Seocheon 33662, Korea; 2 Division of Science Education, Kangwon National University, Chuncheon 24341, Korea; 3 Aqua Team, Aqua Planet Yeosu, Yeosu 59744, Korea; 4 National Marine Bio-Resources and Information Center, National Marine Biodiversity Institute of Korea, Seocheon 33662, Korea; 5 Ocean@Fish Research, Busan 46061, Korea

**Keywords:** artificial breeding, restoration, satellite tracking, sea current, travel path

## Abstract

**Simple Summary:**

Despite the continuous observation and drift of globally endangered sea turtles in Korean waters, relevant research and protection policies are deficient. To restore sea turtle populations near Korea, the Ministry of Oceans and Fisheries, Republic of Korea, has been hatching and rearing sea turtles in captivity and releasing juveniles into the sea. We satellite-tracked juvenile loggerhead and green turtles to confirm their movement patterns and adaptability in the wild. The tracked sea turtles used sea currents for movement, and the tracking duration and movement patterns differed according to their body size. In addition, while the loggerhead turtles moved to the Northeast East Sea, the green turtles tended to move west or southwest from the release point. By considering the release time and location, according to the growth level and species, the adaptability of artificially hatched and reared turtles to natural habitats is expected to be high.

**Abstract:**

With most sea turtle populations declining, activities to conserve their habitat and nesting grounds and restore their populations are being implemented worldwide. To preserve the Northwestern Pacific populations, the National Marine Biodiversity Institute of Korea has been releasing artificially propagated sea turtles, but whether these individuals join the wild population remains unknown. The present study aimed to determine the movement patterns of artificially propagated juvenile loggerhead (*Caretta caretta*) and green (*Chelonia mydas*) turtles fitted with satellite transmitters on their carapaces and released in the waters of Jeju or Yeosu, Republic of Korea, between August 2018 and April 2022. Loggerheads traveled northward to the East Sea, whereas green turtles moved west or southwest. Two 36-month-old and two 48-month-old loggerheads moved toward their potential nursery grounds and toward their feeding grounds, respectively. Three green turtles with a curved carapace length (CCL) of <40 cm moved toward their nursery or feeding grounds, while three individuals (CCL > 45 cm) moved toward their inshore foraging areas. The travel paths were closely related to the direction of local sea currents. Our results implied that releasing artificially propagated sea turtles, considering their age and CCL, can positively contribute to the conservation of Northwestern Pacific populations.

## 1. Introduction

Sea turtle populations are declining worldwide as a consequence of bycatch, overfishing by humans, habitat loss, disease, climate change, and marine pollution [1,2,3,4,5]. For these reasons, all seven known sea turtle species have been designated as Appendices I species in the Convention on International Trade in Endangered Species of Wild Fauna and Flora (CITES). In addition, the International Union for Conservation of Nature (IUCN) has classified six turtle species as critically endangered (CR), endangered (EN), or vulnerable (VU), except for the flatback turtle (*Natator depressus*; Garman, 1880), for which data are insufficient [6]. Sea turtles are protected by various laws in many local governments and countries [7]. Moreover, to conserve or recover sea turtle populations, various conservation and restoration projects are being implemented, including feeding and nursery habitat conservation [8], nesting ground conservation [9], and release after artificial propagation [10].

Five sea turtle species have been reported from the coasts of the Republic of Korea, including the loggerhead (*Caretta caretta*; Linnaeus, 1758), green (*Chelonia mydas*; Linnaeus, 1758), hawksbill (*Eretmochelys imbricata*; Linnaeus, 1766), olive ridley (*Lepidochelys olivacea*; Eschscholtz, 1829), and leatherback (*Dermochelys coriacea*; Vandelli, 1761) turtles [11,12,13,14,15]. The observation frequency of sea turtles is increasing near the Korean Peninsula [13,16], with up to 30 observations reported in one year, of which loggerhead and green turtles were the most frequently observed [11,12,16]. In particular, approximately 10 cases of loggerhead turtle nesting have been reported previously, with one successful hatching case on Jeju Island [5,11,12,16,17]. However, there are still few individual records and limited research on the conservation of sea turtles near the Korean Peninsula. Considering the degree and speed of recent climate change [18,19], ecological studies of sea turtles occurring in Korean waters, at the outer boundary of their distribution, are urgently needed, especially for the conservation of loggerhead and green turtles. Thus, in 2012, the Ministry of Oceans and Fisheries, Republic of Korea, has designated sea turtles as marine protected species and is also conducting restoration projects that preserve stranded sea turtles in the nearby sea and release artificially hatched and reared sea turtles into the wild [20].

The release of juvenile or sub-adult sea turtles after artificial breeding has often been used to conserve and recover sea turtle populations [10,21,22]. As the mortality rate of newborn sea turtles in nature is very high [23], the release of grown juveniles or sub-adults may contribute to increasing their survival rate [9,24]. To evaluate the success of release projects, it is necessary to determine whether the individuals released after artificial breeding successfully join the natural feeding or breeding population. Previous studies have been conducted to verify the origin of sea turtles in a given population by using molecular analysis of individuals captured in the feeding and breeding grounds and by satellite tracking individuals released at hatchling sites with tagged transmitters [25]. The results of such studies can provide information for understanding the life history and population connectivity of the studied species [26]. Although molecular methods are increasingly used [27,28], satellite tracking is still a useful method at the outer boundaries of sea turtle distribution, where only a few individuals are available for the study.

In the present study, using satellite tracking, we investigated the movement patterns of juvenile loggerhead and green turtles that were artificially bred and reared in captivity in the Republic of Korea. Additionally, we aimed to evaluate whether releasing sea turtles in the waters of the Republic of Korea contributes to their conservation and restoration in the Northwestern Pacific.

## 2. Materials and Methods

### 2.1. Animals and Site

The National Marine Biodiversity Institute of Korea released 101 juvenile sea turtles, namely 13 loggerhead and 98 green turtles, into the waters of the Republic of Korea between September 2017 and August 2021. All the green turtles were artificially born and reared in captivity at Aqua Planet Yeosu, Republic of Korea. Loggerhead turtles were artificially born in the Port of Nagoya Public Aquarium, Japan, and after being transported to Aqua Planet Yeosu, they were also released into the wild. Most of the turtles were released at Jungmun Beach, Seogwipo, Jeju Island (33.244913° N, 126.412755° E), where fishery and sport fishing activities are low, in order to minimize the accidental catching of turtles in fishing nets. Eight loggerhead turtles were released offshore in the Korea Strait (34.323833° N, 127.86915° E). Before their release, we measured the curved carapace length (CCL, cm), curved carapace width (CCW, cm), and body weight (BW, kg) of all released sea turtles. We tagged the Inconel tag on the flipper for future identification (Table 1).

### 2.2. Satellite Tracking

To determine the movement patterns of the released juvenile sea turtles, we satellite tracked 17 turtles—six loggerhead and 11 green turtles. We used three types of standard back-mount transmitters (Wildlife Computers Inc., Redmond, WA, USA) depending on the body weight of the turtles; SPOT-311 (lifespan: approximately 150 days; 51 × 27 × 19 mm; 40 g) or SPOT-387 (lifespan: approximately 171 days; 72 × 56 × 23 mm; 39 g) was applied to turtles weighing less than 15 kg, and SPOT-352A (lifespan: approximately 920 days; 72 × 56 × 22 mm; 129 g) to turtles over 15.1 kg (Table 1). SPOT-387 is the new version of SPOT-311. We attached the transmitter to the turtle’s carapace with silicone elastomer and epoxy glue after softening the shell scales with sandpaper [29]. To observe the transmitter signal, we used the Argos system (CLS America, http://www.clsamerica.com, accessed on 5 May 2022), which has been effectively and frequently used for sea turtle satellite tracking [30,31,32,33]. To increase the reliability of the tracked data, we used only coordinates with the smallest error radius on a day. In addition, considering the maximum speed of the sea turtles’ movement, when their movement was faster than 5 km/h (100 km/d), the data were excluded from the analysis [32,33,34,35].

We analyzed data only from individuals that were tracked for more than 30 days. Satellite tracking was terminated when the signals from the transmitter were no longer detected by an Argos satellite for more than five months. The movement distance was set as the shortest straight line between the sequential tracked points of the turtles. The total distance moved was determined as the sum of the movement distances from the release site to the last tracking location, where tracking was terminated. The daily distance moved was determined as the distance between the next day’s location and the previous day’s location. We used ArcMap v.10.1 (ESRI, Redlands, CA, USA) to calculate the distance moved and to construct the turtle tracking map [36].

## 3. Results

### 3.1. Satellite Tracking

Of the eight turtles (four loggerhead and four green turtles) were tracked for more than 30 days (Table 1). Four turtles were excluded from the dataset because their transmitter signal failed immediately after release. Of the remaining thirteen turtles, five with a CCL less than 40 cm were tracked for an average of 46.0 ± 47.5 days and only two were tracked for more than 30 days. Eight turtles with a CCL over 40 cm were tracked for an average of 86.0 ± 70.6 days, among which six were tracked for more than 30 days. Satellite tracking of the four loggerhead turtles was carried out between 29 August 2018 and 28 February 2020. At the time of release, KOR0094 and KOR0096 were 36 months old, with CCLs of 44.0 and 38.5 cm, respectively, and KOR0108 and KOR0109 were 46 months old, with CCLs of 55.6 and 52.1 cm, respectively (Table 1). They were tracked for an average of 89.3 ± 32.2 times over 150.5 ± 62.4 days, with an average total distance moved of 2592 ± 197 km, an average straight distance from the release site of 1008 ± 592 km, and an average daily distance moved of 27 ± 7 km.

Satellite tracking of the four green turtles was carried out between 29 August 2018 and 28 April 2022. At the time of release, KOR0101 was 16 months old, with a CCL of 27.1 cm, and KOR0139 was 39 months old, with a CCL of 45.1 cm; KOR0152 and KOR0153 were 52 months old, with CCLs of 58.4 and 55.8 cm, respectively (Table 1). The individuals were tracked for an average of 106.3 ± 58.1 times over 123.8 ± 73.2 days, with an average total distance moved of 3297 ± 2340 km, an average straight distance of 1649 ± 1123 km from the release site, and an average daily distance moved of 27 ± 8 km.

### 3.2. Movement Patterns

**Figure 1:**
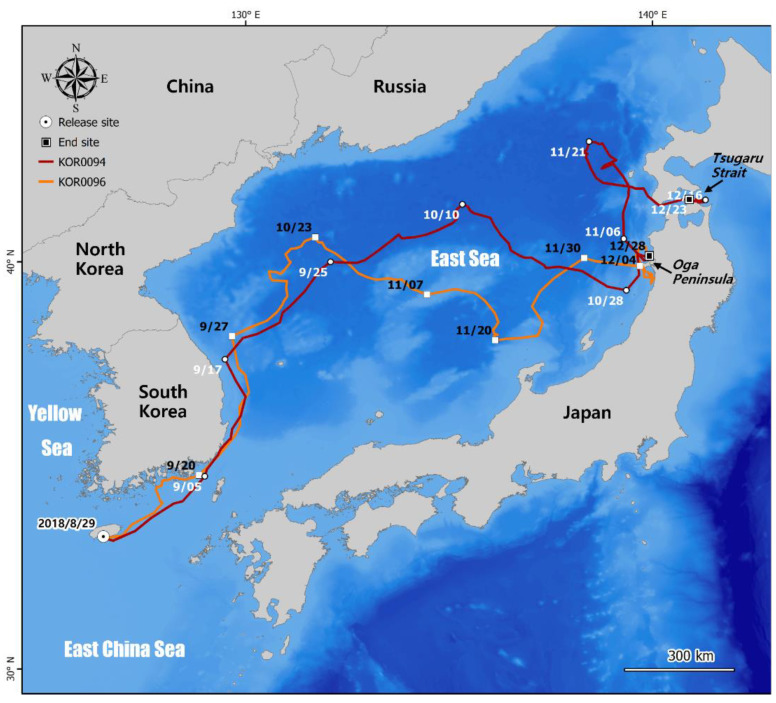
Travel routes of satellite-tracked loggerhead turtles KOR0094 and KOR0096.

All four loggerhead turtles initially headed northeast and moved into the East Sea, regardless of the release location (Table 2). Thirty-six-month-old KOR0094 and KOR0096 showed a similar movement pattern, and they both finally traveled to the vicinity of the Tsugaru Strait, Japan, where they could move to the Pacific Ocean. Specifically, KOR0094 moved into the East Sea approximately one week after its release at Jeju Island, after which it moved further north along the east coast of the Korean Peninsula until 17 September, and then crossed the East Sea to the Oga Peninsula (39.84363° N, 139.653107° E) over approximately 44 days. It was last identified in the vicinity of the Tsugaru Strait (41.54370° N, 140.91959° E), which was 1577 km away from its release site. KOR0096 moved more slowly than KOR0094; after moving northward along the east coast, it took approximately 70 days to cross the East Sea and arrive near the Oga Peninsula. Its last tracking point was from the neighboring sea of the Oga Peninsula (40.15199° N, 139.91525° E), which was 1427 km away from its release site (Figure 1).

**Figure 2:**
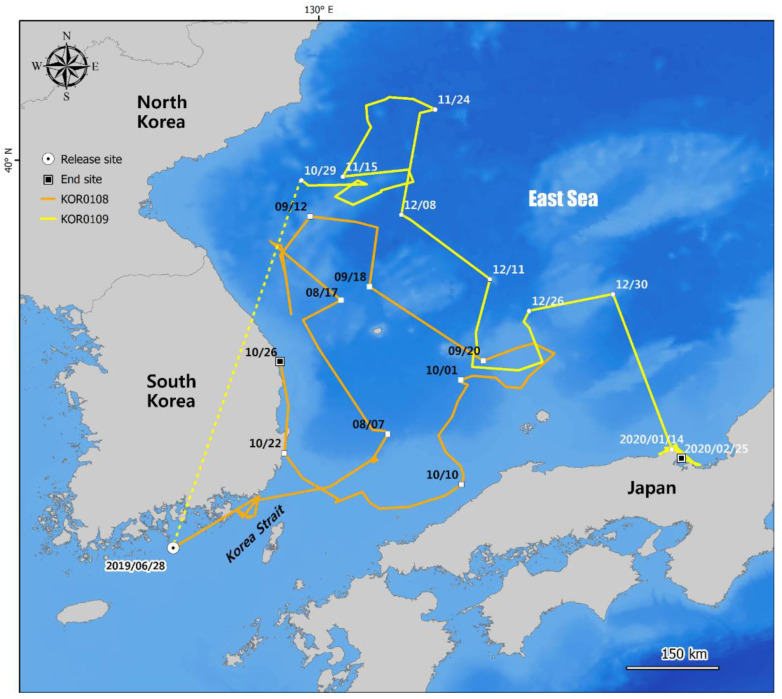
Travel routes of two satellite-tracked loggerhead turtles, KOR0108 and KOR0109. In the case of KOR0109, the dotted line between the release date and 10/29 (29 October) indicates a period when no tracking signal was received; the reasons for this are unknown.

Both 46-month-old loggerhead turtles, KOR0108 and KOR0109, were initially oriented toward and moved into the East Sea. KOR0108 moved north to 39.17964° N, 129.87413° E, along the east coast of the Korean Peninsula, after which it turned southward to the open sea near the Shimane Prefecture, Japan (35.25219° N, 132.08944° E), and was finally recorded at the coast of the East Sea, Uljin, Gyeongsangbuk-do, Republic of Korea (37.04580° N, 129.43513° E), which was 333.6 km away from its release site (Figure 2). KOR0109 showed a similar movement pattern. It initially moved northward to the open sea (40.93005° N, 131.03068° E), about 100 km northeast of Hamgyeongbuk-do, North Korea, and then turned southward to the open sea near the Kyoto Prefecture, Honshu, Southern Japan, over three months. This individual was finally identified as a corpse on 17 May 2020, on a beach in the Iwagahana, Kyoto, Japan (35.65478° N, 135.25809° E), 5.2 km away from its last tracking point (Figure 2).

**Figure 3:**
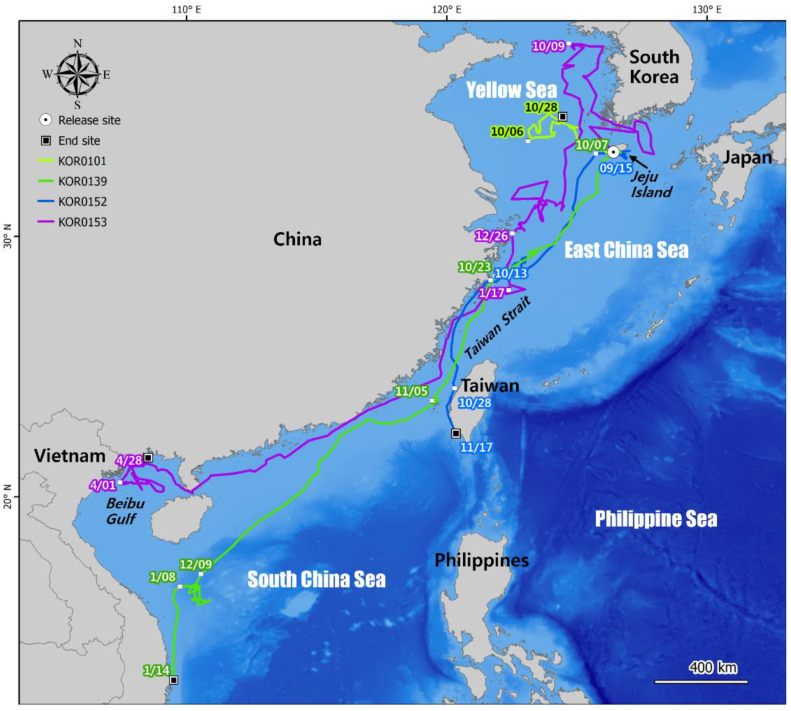
Travel routes of four satellite-tracked green turtles. KOR0101 with a CCL less than 40 cm stayed in the Yellow Sea, while three turtles with CCLs over 40 cm traveled to the southwest.

During the satellite tracking of green turtles, one individual moved around the Jeju Sea, while three of them moved to the southwest (Figure 3). KOR0101 initially moved toward the west parts of Jeju Island and was finally confirmed in the vicinity of Hongdo in the Yellow Sea, Republic of Korea (34.59641° N, 124.42068° E), 237.5 km away from its release site. KOR0139, KOR0152, and KOR0153 moved to the Taiwan Strait, but then each moved in a different direction. KOR0139 remained near Jeju Island for 26 days from the date of release and then moved southwestward along the east coast of China. In early December, it arrived near Paracel Islands in the South China Sea (16.48141° N, 110.29719° E) and stayed in that area for approximately one month. It was finally tracked in the open sea near Dong Hoa, Phu Yen Province, Vietnam (12.97541° N, 109.50287° E), 2829 km away from its initial release site. KOR0152 stayed near Jeju Island for 18 days after its release and then moved southwest. After 43 days of travel, KOR0152 arrived near Changhua County (24.16678° N, 120.30350° E), Taiwan. While it was moving south along the coast of Taiwan, on 17 November, tracking was terminated near Kaohsiung (22.45660° N, 120.35819° E), 1335 km away from the release point. KOR0153 moved to the north of the Yellow Sea for about 44 days from the day of release, and then, from October 10, it started moving south along the eastern coast of China to the Beibu Gulf (20.54729° N, 107.45156° E) near Quang Ninh, northeast of Vietnam, 2344 km away from the release point at the end of 1 April 2022. After that, it did not move further south and stayed at the Beibu Gulf until the tracking ended.

## 4. Discussion

Our results show that the CCL of the released sea turtles should be approximately 40 cm or longer to obtain data on a reasonable movement path. In previous studies, the mean tracking period of juvenile loggerhead turtles with a CCL of less than 20 cm was 70 days, and that of green turtles was 66 days [37,38]. In the present study, it seems that the long-term tracking of the loggerhead turtle KOR0096 was possible because, even though the CCL was less than 40 cm, it was close to this size (38.5 cm). On the other hand, out of seven green turtles with a CCL less than 40 cm, only KOR0101 was tracked for 61 days. This shows that long-term satellite tracking is not appropriate for sea turtles with a CCL less than 40 cm, especially in green turtles. Two factors may be responsible for short tracking periods. First, in general, the detachment of transmitters from satellite-tracking sea turtles occurs frequently when they are young because the carapace of young sea turtles expands rapidly and molting occurs frequently due to rapid growth [37,39]. Second, the high mortality rate of juvenile sea turtles may also have contributed to the shorter satellite tracking periods for juvenile sea turtles [40]. Therefore, we recommend that a CCL of 40 cm is the minimum appropriate size for long-term tracking. Recently, many studies have indirectly tracked the travel path of sea turtles by using genetic analysis of individuals appearing in feeding and breeding areas [3,5]. Nevertheless, satellite tracking is still a useful study method in the outer boundary regions where sea turtles occasionally appear. Therefore, when conducting a satellite tracking study of sea turtles, appropriate consideration of subject size and age is important for accurate data acquisition.

### 4.1. Movement Patterns of Loggerhead Turtles

Most sea turtles migrate long distances, but movement patterns differ among species, as well as within the same species, depending on their life stage [41]. In this study, artificially reared loggerhead and green turtles also had distinct movement patterns. All the loggerhead turtles traveled northward to reach the East Sea, whereas the green turtles all moved to the west or southwest from their release sites. The orientation of loggerhead turtles toward the East Sea coincided with the migration of sea turtles hatched in the wild populations [42]. In the Northwestern Pacific, nesting sites of loggerhead turtles range from the Kanto District to the shoreline of the Ryukyu Archipelago, Japan [3,43,44,45]. Loggerhead turtles naturally hatched in Japan move into the North Pacific Ocean via the northeasterly Kuroshio Current and the Kuroshio Extension Current [46,47,48,49]. They then cross the Pacific Ocean to California or Mexico, where they spend their juvenile periods [27,50,51,52]. Recent satellite tracking of immature loggerheads hatched and reared in captivity also showed similar movement paths to those of wild loggerheads [42]. Therefore, the northeast orientation of loggerhead turtles released into the East Sea, regardless of the release points, was consistent with these previous findings.

The detailed movement patterns of loggerhead turtles are likely dependent on the age of the released turtles. The 36-month-old KOR0094 and KOR0096 migrated to the north part of Honshu, near the Tsugaru Strait, by crossing the East Sea, and their orientation was similar to that of naturally hatched juvenile loggerheads. On the other hand, 46-month-old KOR0108 and KOR0109 were initially oriented to the northeast but did not cross the East Sea. Instead, they changed their travel path toward the south at the central part of the East Sea and continued straight down to the southern coast of Honshu. To further understand why the 46-month-old sea turtles showed different movement patterns to the 36-month-olds, we further examined their body sizes. After the CCL of juvenile loggerheads reaches 50–75 cm, they return from the Eastern Pacific region, where they mainly reside, to their nesting grounds in Japan [46,53,54,55]. Loggerhead turtles with a CCL of 75 cm or higher participate in breeding [56,57]. Sub-adult loggerhead turtles often cross currents to reach their desired destinations [52,58]. Considering that the CCLs of 46-month-old KOR0108 and KOR0109 were 55.6 and 52.1 cm, respectively, they can be expected to move directly to the feeding grounds for adults instead of to nursery grounds for sub-adults. Their relatively large body size allows them to move against the northeast direction of the East Korean Warm Current (EKWC) in the East Sea, resulting in farther movement south and returning to Kyushu, Japan’s southern coast.

**Figure 4:**
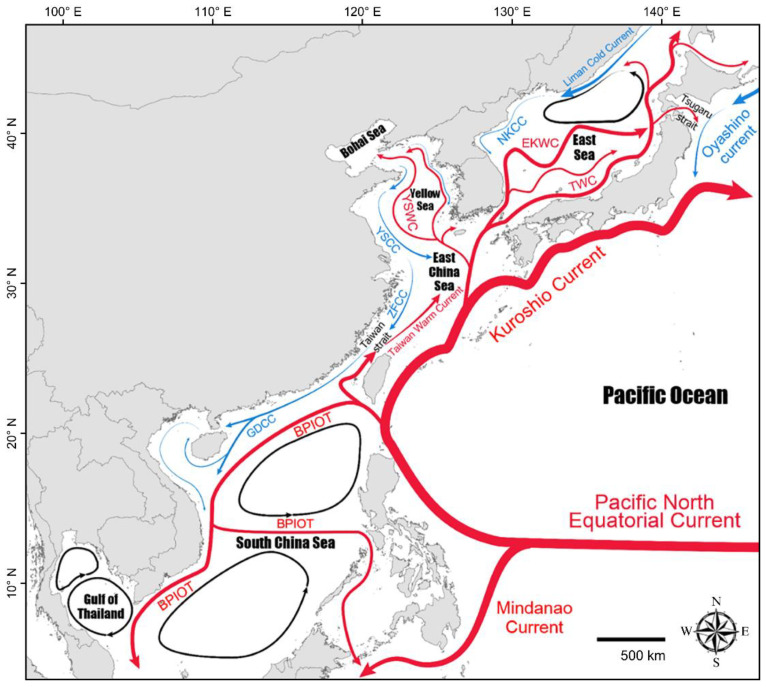
Topography and typical current patterns in the North Pacific Ocean (with reference to References [61,62,63]). Red arrows indicate warm currents, blue arrows indicate cold currents, and black arrows indicate gyres. BPIOT, Branch of the Pacific-to-Indian Ocean Throughflow; EKWC, East Korean Warm Current; GDCC, Guangdong Coastal Current; NKCC, North Korea Cold Current; TWC, Tsushima Warm Current; YSCC, Yellow Sea Coastal Current; ZFCC, Zhejiang-Fujian Coastal Current.

The travel paths of 36-month-old turtles were similar to the directions of the ocean currents flowing through the East Sea. For example, the EKWC flows in a northeast direction through the East Sea, and the paths of KOR0094 and KOR0096 crossing the East Sea were very similar to each other (Figure 4), implying that both individuals crossed the East Sea on the EKWC. When compared to late juveniles and sub-adults, the movement pattern of hatchlings and early juvenile sea turtles is more dependent on sea currents [41,52,59]. If the individuals that arrived at the northern part of Honshu survived, they would pass through the Tsugaru Strait, ride the Oyashino Current, be transported by the Kuroshio Current, and move to the east Pacific Ocean, where they would spend their juvenile periods. Saito et al. [42] reported that most juvenile loggerheads with a straight carapace length (SCL) of less than 40 cm that were released at Kanazawa, Western Japan, moved into the Pacific Ocean via either the Soya Strait or Tsugaru Strait, while the majority of loggerheads with a SCL of 60 cm or larger moved northward and stayed near the release site. The movement of 46-month-old KOR0108 and KOR0109 also partially coincided with the trajectory of the southward ocean current, where the EKWC met the North Korean Cold Current (NKCC), moving northward and then temporarily moved southward. Afterward, these turtles might migrate southward by actively swimming against the EKWC in areas with a relatively weak current strength [60].

### 4.2. Movement Patterns of Green Turtles

The orientation of juvenile green turtles can be affected by the location of their natural nursery and feeding grounds and the direction of ocean currents. In the wild, the orientation of green turtle juveniles with a CCL of 40 cm or less is passively determined by ocean currents. When they become larger juveniles with a CCL over 40 cm, they migrate from pelagic nursery habitats to coastal feeding areas [64]. In this study, KOR0101 was the only young turtle with a CCL less than 40 cm and stayed for 61 days in the middle of the Yellow Sea, not off the coast. The differentiated movement pattern of KOR0101 is unlikely to be caused by the shortest tracking period, given that the other three turtles showed a clear southward movement along the coast of Korea or China. The feeding sites of green turtles in the Northwestern Pacific are widely distributed, from the east coast of Honshu to the southern coast of Shikoku, through the Ryukyu Archipelago, Japan, to Taiwan [65,66,67]. Therefore, the green turtles released in the present study might simply react passively to the flow of sea currents or actively move toward a possible nearby feeding site, as reported previously [28,68]. KOR0101 moved westward after being released from Jeju Island and may have been passively driven by the Yellow Sea Warm Current (YSWC), or it may have moved in search of possible nursery or feeding sites in the Yellow Sea. Since green turtles are frequently observed in the waters near the Korean Peninsula, and sightings consist of many relatively young individuals [16], it is necessary to carry out the origin tracking studies for green turtles by using movement analysis in the future.

KOR0139, KOR0152, and KOR0153 might have moved in search of foraging habitats as sub-adults. Adult green turtles often migrate long distances to return to their natal breeding sites [69,70], but KOR0139, KOR0152, and KOR0153, whose CCLs were over 45 cm, were between the post-juvenile and reproductively mature adult stages, considering that green turtles begin breeding when their CCL reaches approximately 80 cm [64]. When green turtles grow to approximately 44 cm CCL, they leave the pelagic area used as their nursery feeding site and move into the inshore areas as their novel foraging site [71,72]. Thus, these three turtles would have a distinct movement pattern compared to KOR0101 with a smaller CCL. In the Western Pacific, the foraging habitats of juvenile green turtles are mainly located in tropical and subtropical areas of Malaysia [66,73]. Sea currents serve as efficient transport systems for green turtles [65], and our results support this. If KOR0139, KOR0152, and KOR0153 used the coastal, southward-running Zhejiang-Fujian Coastal Current (ZFCC) for migration, passive and active southward movement would be possible [74,75,76]. The actual travel routes of KOR0139, KOR0152, and KOR0153 were similar to the moving direction of this coastal current. It is assumed that KOR0139 and KOR0153 traveled beyond Taiwan to the Paracel Islands in the South China Sea along the Guangdong Coastal Current, after which they migrated to the east coast of Vietnam. The tracking of KOR0152 stopped during its southward journey along the coast of Taiwan, but when the location and directions of the Branch of the Pacific-to-Indian Ocean Throughflow (BPIOT) are considered, it is assumed that KOR0152 would not have continued to travel south to the Philippines. Although Taiwan is a feeding site for green turtles [65], KOR0152 did not stop moving while the satellite tracker was working. Considering the travel route of KOR0139 and KOR0153, it is possible that KOR0152 could also have headed to Vietnam on the BPIOT. The reason for such a movement pattern might be related to the sea temperatures. Considering their departure near the release site in the fall, between mid-September and early October, these turtles might further migrate southward in search of a habitat with a warmer temperature range. KOR0139 was released in the fall and moved south four days after release. After KOR0152 and KOR0153 were released in late summer (August), they stayed near Jeju Island and the west coast of the Korean Peninsula, respectively, and then started to travel south in early October. Further research is needed to determine whether this movement pattern is typical of subadult green turtles with a CCL of 40 cm or larger.

## 5. Conclusions

In summary, both juvenile loggerhead turtles and green turtles hatched in captivity showed a tendency to migrate toward the nursery or feeding habitats that naturally hatched turtles in the wild population do. When the CCL of the released sea turtles was larger, the follow-up tracking period was longer, and a clearer movement pattern was observed. These results indicate that the release of artificially propagated individuals, with consideration of their age and size, can have a positive effect on natural populations. Although it was not possible to confirm their final destination because of the small number of sea turtles released and the relatively short satellite tracking period, we conclude that the release of sea turtles hatched and reared in captivity in the Republic of Korea could contribute to the conservation of the wild populations in the neighboring Western North Pacific.

## Figures and Tables

**Table 1:** Individual information and characteristics of *Caretta caretta* and *Chelonia mydas* satellite tracked and analyzed in the present study. The turtles whose transmitter signal had been cut off immediately after release are marked with ‘-’ in ‘Tracking Days’. CCL = curved carapace length; CCW = curved carapace width.

Species	Turtle ID	Hatching Date	Release Date	Age (Months)	CCL (cm)	CCW (cm)	Body Weight (kg)	Tracking Days	Used in Analysis
*Caretta caretta*	KOR0093	Aug 2015	29 Aug 2018	36	42.0	36.0	14.8	10	X
	KOR0094	Aug 2015	29 Aug 2018	36	44.0	37.0	14.6	115	O
	KOR0095	Aug 2015	29 Aug 2018	36	43.0	36.0	14.4	8	X
	KOR0096	Aug 2015	29 Aug 2018	36	38.5	34.0	12.7	122	O
	KOR0108	Aug 2015	28 Jun 2019	46	55.6	45.7	26.5	121	O
	KOR0109	Aug 2015	28 Jun 2019	46	52.1	44.1	23.8	244	O
*Chelonia mydas*	KOR0098	Apr 2017	29 Aug 2018	16	24.8	23.0	2.48	13	X
	KOR0099	Apr 2017	29 Aug 2018	16	24.5	21.1	2.21	6	X
	KOR0100	Apr 2017	29 Aug 2018	16	25.6	22.5	2.62	-	X
	KOR0101	Apr 2017	29 Aug 2018	16	27.1	23.4	2.67	61	O
	KOR0123	Apr 2017	28 Aug 2019	28	37.4	30.9	6.2	-	X
	KOR0124	Apr 2017	28 Aug 2019	28	35.2	30.3	5.7	-	X
	KOR0137	Jun 2017	11 Sep 2020	41	36.9	31.0	6.3	28	X
	KOR0138	Jun 2017	11 Sep 2020	41	43.0	39.3	9.1	-	X
	KOR0139	Jun 2017	11 Sep 2020	41	45.1	35.0	10.2	125	O
	KOR0152	Apr 2017	26 Aug 2021	52	58.4	51.9	22.1	83	O
	KOR0153	Apr 2017	26 Aug 2021	52	55.8	48.8	18.5	226	O

**Table 2:** Summary of the satellite-tracking of *Caretta caretta* and *Chelonia mydas*.

Species	Turtle ID	Release Location	Final Location	Tracking			Traveled Distance (km)	
				Period	Days	Numbers	Total	Mean Daily
*Caretta caretta*	KOR0094	Jeju	41.54370° N 140.91959° E	29 Aug. 2018–23 Dec. 2018	115	113	2885	30.6 ± 24.1
	KOR0096	Jeju	40.15199° N 139.91525° E	29 Aug. 2018–28 Dec. 2018	122	121	2519	24.9 ± 20.4
	KOR0108	Yeosu	37.04580° N 129.43513° E	28 Jun. 2019–26 Oct. 2019	121	62	2510	35.3 ± 34.9
	KOR0109	Yeosu	35.61106° N 135.20114° E	28 Jun. 2019–28 Feb. 2020	244	61	2455	18.7 ± 22.3
*Chelonia mydas*	KOR0101	Jeju	34.59641° N 124.42068° E	29 Aug. 2018–28 Oct. 2018	61	60	909	18.1 ± 13.1
	KOR0139	Jeju	12.97541° N 109.50287° E	11 Sep. 2020–14 Jan. 2021	125	110	3838	34.5 ± 35.2
	KOR0152	Jeju	22.45610° N 120.35793° E	26 Aug. 2021–17 Nov. 2022	83	68	6309	30.4 ± 30.0
	KOR0153	Jeju	21.50072° N 108.45682° E	26 Aug. 2021–28 Apr. 2022	226	187	2134	29.4 ± 23.2

## Data Availability

Not applicable.
